# Hydrogen sulfide upregulated mRNA expressions of sodium bicarbonate cotransporter1, trefoil factor1 and trefoil factor2 in gastric mucosa in rats

**Published:** 2016-12-15

**Authors:** Parisa Cheraghi, Seyyed Ali Mard, Tahereh Nagi

**Affiliations:** 1 Department of Molecular and Cellular Sciences, Faculty of Advanced Sciences and Technology, Pharmaceutical Sciences Branch, Islamic Azad University, Tehran, Iran; 2 Research Center for Infectious Diseases of Digestive System, Physiology Research Center, Department of Physiology, School of Medicine, Ahvaz Jundishapur University of Medical Sciences, Ahvaz, Iran

**Keywords:** NaHS, Propargylglycine, Sodium bicarbonate cotransporter 1, Trefoil factor1, Trefoil factor2

## Abstract

Hydrogen sulfide (H_2_S) has been shown to protect the gastric mucosa through several protective mechanisms but till now its effect on mRNA expression of sodium bicarbonate cotransporter 1 (NBC1), trefoil factor1 (TFF1) and trefoil factor2 (TFF2) was not investigated. This study was aimed to evaluate the effect of H_2_S on mRNA expression of NBC1, TFF1 and TFF2 in rat gastric mucosa in response to gastric distention. Thirty two rats were randomly assigned into four equal groups. They were control (C), distention (D), propargylglycine (PAG)-, and NaHS-treated groups. To evaluate the effect of exogenous and endogenous H_2_S on gene expression of NBC1, TFF1 and TFF2, two groups of rats were received H_2_S donor, intra-peritoneal NaHS (80 µg Kg^-1^), and PAG (50 mg kg^-1^), accompanied to stimulate the gastric acid secretion, respectively. Under general anesthesia and laparotomy, a catheter was inserted into the stomach through duodenum for instillation of isotonic saline for gastric distention. Ninety min after beginning the experiment, animals were sacrificed and the gastric mucosa was collected to determine total acid content of gastric effluents and to quantify the mRNA expression of studied genes by quantitative real-time polymerase chain reaction (qRT-PCR). Results showed that A) gastric distention increased the level of mRNA expressions of NBC1, TFF1 and TFF2; B) these levels in NaHS-treated rats were significantly higher than those in Distention group; and C) PAG decreased the expression levels of NBC1 and TFF1. The Findings showed H_2_S upregulated gene expression of NBC1, TFF1 and TFF2 in gastric mucosa.

## Introduction

Hydrogen sulfide (H_2_S) has been shown to protect the gastric mucosa against noxious factors such as gastric acid through several protective mechanism.^1^ Hydrogen sulfide endogenously is produced by enzymatic and non-enzymatic pathways. Two key enzymes, cystathionine gamma lyase (CSE) and cystathionine beta synthase (CBS) are involved in endogenous generation of H_2_S. They convert L-cysteine to hydrogen sulfide in mammalian body.^2^^,^^3^ It has been shown that both CBS and CSE are expressed in the gastric mucosa in rat but the main responsible enzyme for H_2_S production is CSE.^2^^,^^3^ The gastric mucosal barrier consists of three layers: pre-epithelial, epithelial and sub-epithelial barriers.^4^ The H_2_S has been shown to contribute in sub-epithelial barrier by increasing gastric mucosal blood flow and also promote the pre-epithelial barrier by stimulation of mucus and bicarbonate secretion.^5^ Moreover, exogenous and endogenous H_2_S has been shown to decrease the gastric acid output in response to distention-induced gastric acid secretion.^6^^,^^7^

It is well known that the gastric mucosa is continuously exposed to many noxious factors such as gastric acid, pepsin and ingested materials. How the structural integrity of gastric mucosa maintains and resists against 100 mM HCl (gastric acid) has puzzled investigators for more than 200 years. The idea that presence of continuous circulation of alkaline blood through the mucosa neutralizes acid was the first hypothesis suggested by Hunter.^8^ The first line of mucosal defense, pre-epithelial barrier is formed by mucus gel, bicarbonate and surfactant phospholipids, which cover the mucosal surface. A continuous bicarbonate secretion by surface epithelial cells produces and maintains a neutral microenvironment (pH 7.0) at the surface epithelial cells. At this pH, gastric acid neutralizes and pepsin denatures.^8^ Therefore, these noxious factors cannot penetrate and affect the surface mucus cells. Epithelial cells secret bicarbonate along with mucus. Some bicarbonate is formed intracellularly from H_2_O and CO_2_ but the main origin is the uptake from interstitial fluid. It has been shown that Na^+^-HCO_3_^-^ co-transporter (NBC) at basolateral membrane of epithelial cells act as the major mechanism for import of bicarbonate.^9^ It has been shown that H_2_S stimulate bi-carbonate secretion in duodenum and stomach in the rat.^5^

The trefoil proteins (TFFs) are synthetized and secreted by mucosal epithelia.^10^^,^^11^ There are three types of these proteins: TFF1 and TFF2 are constitutively expressed in stomach and the third isoform (TFF3 or intestinal type of TFF, ITF) is mainly expressed in goblet cells of the small and large intestine.^10^^,^^11^ These peptides are contributed in the mechanisms of defense and repair by interacting with mucins to form the mucus barrier and promote the process of restitution.^12^ Trefoil proteins have been shown to enhance mucosal barrier functions by stabilizing the mucus gel and promoting epithelial restitution.^12^^,^^13^ These small protease-resistant proteins also have been shown to protect the gastric mucosa against NSAIDs-, and stress-induced gastric erosions in mice and rats.^14^^,^^15^

To our knowledge, there is no study about the effect of endogenous and exogenous H_2_S on gene expression of NBC1, TFF1 and TFF2. Thus, this study was aimed to evaluate the effect of H_2_S on mRNA expression of NBC1, TFF1 and TFF2 in rat gastric mucosa in response to gastric distention.

## Materials and Methods

**Animals. **Male Wistar rats (150-200g) were supplied from the animal house of Ahvaz Jundishapur University of Medical Sciences, Ahvaz, Iran. Animals were fed on conventional diets and tap water *ad libitum*. They were maintained under standard conditions of humidity, temperature (22.0 ± 2.0 ˚C) and light/dark cycle (12 hr: 12 hr). All experiments were carried out in accordance with ethics committee of Ahvaz Jundishapur University of Medical Sciences, Ahvaz, Iran.

**Animal grouping and surgical procedures. **Thirty-two male Wistar rats (150 to 200 g) were randomly assigned into four groups (n = 8 per group). They were control (C), distention (D), propargylglycine (PAG; a selective inhibitor of cystathionine gamma lyase)-, and NaHS (a H_2_S donor)-treated groups. Fasted rats were anesthetized using an intraperitoneal injection of 60 mg kg^-1 ^ketamine (Alfasan, Woerden, The Netherlands) and 15 mg kg^-1 ^xylazine (Alfasan) combination. Depth of anesthesia was monitored throughout the experiment by the pedal withdrawal (toe pinch) reflex every 30 to 45 min. If the reﬂex was observed, a supplemental dose of anesthetics (one third of initial dose) was administered to maintain adequate anesthesia. Animals’ body temperature were controlled with a rectal thermometer and maintained at 37.0 ± 0.5 ˚C using a homeothermic blanket control system (Harvard Apparatus Ltd., Edenbridge, UK). To maintain a patent airway, anesthetized rats underwent tracheostomy. After a midline laparotomy, both the stomach and the duodenum were exposed. A polyethylene catheter (3.0 mm outer diameter) was inserted into the stomach through the duodenum and held in place by a ligature around the pylorus. At the beginning of each experiment, the lumen of the stomach was gently rinsed with isotonic saline (pH = 7, 37.0 ˚C) until gastric washout was clear. Thirty min after surgical operation, proper volume of isotonic saline was instilled into the stomach of control and experimental groups and 15 min later the gastric content was washed and new equal volume of isotonic saline was instilled through duodenal catheter into the stomach and repeated until end of the experiment.

Control group: 1 mL of isotonic saline (pH=7 and 37.0 ˚C) was instilled into the stomach.

Distention group: The stomach was distended by isotonic saline (1.5 mL per 100 g of body weight, pH= 7 and 37.0 ˚C).

PAG-, and NaHS-treated groups: Animals were received CSE inhibitor, PAG (50.0 mg kg^-1^, i.p.); and NaHS (H_2_S donor; 80.0 µg kg^-1^, i.p.) along with gastric distention by isotonic saline (1.5 mL per 100 g of body weight, pH = 7 and 37.0 ˚C).^6^

After 90 min of isotonic saline instillation (including six consecutive instillations of isotonic solution), animals were sacrificed by an overdose of anesthetics, their stomachs were removed and gastric contents collected. Acidity and pH of each sample were measured with an autotitrator pH meter (Radiometer, Copenhagen, Denmark) by automatic potentiometric titration to pH 7 with NaOH (0.01 N) and was expressed as µEq H^+^ per 15 min. Total acid output was expressed as µEq H^+^ per 90 min (six consecutive washouts). After collecting the gastric contents for measurement of acidity and pH, the stomachs were opened along the greater curvature, rinsed with physiological saline and pinned out in ice-cold saline. Fifty mg of gastric mucosal tissues were quickly excised, snap-frozen and stored in liquid nitrogen for mRNA analysis.

**RNA extraction and cDNA synthesis. **The total RNA was extracted from the frozen tissue samples using RNeasy Plus Mini Kit (Qiagen, Hilden, Germany). The purity and concentration of the extracted RNA was determined spectrophotometerically at 260 and 280 nm wavelength (BioPhotometer Plus; Eppendorf, Hamburg, Germany). The cDNA was synthesized from one microgram of the total RNA using QuantiTect reverse transcription Kit (Qiagen) according to the manufacturer’s instruction.

**Quantitative real-time PCR. **The mRNA levels of the NBC1, TFF1, TFF2, and the housekeeping gene glycer-aldehyde-3-phosphate dehydrogenase (GAPDH) were measured by quantitative real-time PCR (qRT-PCR) using LightCycler^®^ 480 system (Roche Diagnostics, Branford, USA). The specific primers (Bioneer, Daejeon, South Korea) for measurement of target genes and GAPDH were used and the lengths for amplified products were as follow: GAPDH (5´-TGCTGGTGCTGAGTATGTCGTG-3 and 5´ CGG AGATGA TGACCCTTTTGG-3´, 101 bp); NBC1(5´-CTCACAT TCGGTCGCCAAAC-3´and 5´-CCCCAATGTAGATC GTGT GGT-3, 149 bp); TFF1 (5´-TCCCTTCTAAGGTCCAT CCGA-3´and 5´-CAGATCCA GCCACCGAACAG-3, 126 bp); and TFF2 (5´-GACGC CCTCCAACAG AAAGA-3´and 5´CGACGCTT GGTTTGGAAGT G-3, 133 bp). All PCR amplifications were performed in duplicate reactions and in final volume of 20 µL containing 2 µL cDNA, 0.8 µL of specific primers, 10.0 µL of master SYBR Green Master Mix: SYBR Premix Ex Taq II (Tli RNase H Plus; Takara Bio Inc., Shiga, Japan), 6.4 µL ddH_2_O using the following protocol: Pre-incubation at 95 ˚C for 2 min to activate DNA Taq polymerase and 40 two-step cycles with denaturation with denaturation at 95 ˚C for 20 sec, and annealing/ extension at 60 ˚C for 1 min. In addition, the no-template negative control (H_2_O) was routinely run in every examined at the end of amplification process to ensure the specificity of PCR products. The purity of each amplicon for each reaction was further confirmed by agarose gel electrophoresis (data not shown). Expression level of NBC1, TFF1 and TFF2 were normalized against GAPDH expression (internal calibrator for equal RNA template loading and normalization). To determine the relative quantification of gene expression, comparative cycle of threshold (Ct) method with arithmetic formulae (2^−ΔΔCt^) was used.

**Statistical analysis. **Statistical analysis was performed by one-way ANOVA and followed by *post hoc* Tukey’s test using SPSS (version 17; SPSS Inc., Chicago, USA). Significance was set at a *p < *0.05 level.

## Results

**Fig. 1:**
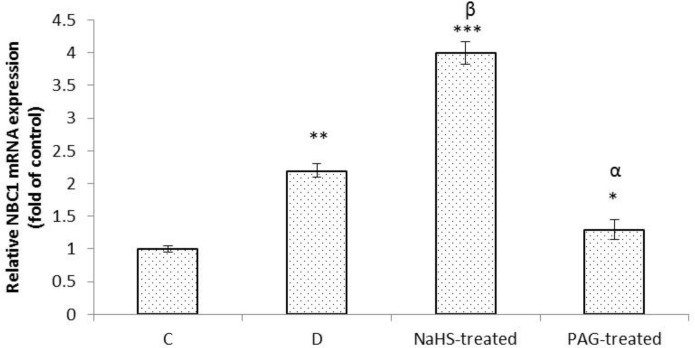
Effects of gastric distention alone and in combination with sodium hydrosulfate (NaHS) or propargilglycine (PAG) on mRNA expression of NBC1 in gastric mucosa. Analysis of qRT-PCR results showed that the level of mRNA expression of NBC1 was increased in response to distention, and NaHS treatment. NaHS-treated: Animals received H_2_S donor (NaHS), PAG-treated: Endogenously production of hydrogen sulfide was inhibited using selective CSE inhibitor (PAG). *, ** and *** indicate significant difference compared to the control group (C) at *p *< 0.05, *p *< 0.01 and *p *< 0.001, respectively. ^α ^indicate significant decrease compared to distention group (D) at *p *< 0.05; ^β ^indicate significant increase compared to distention group at *p *< 0.01. Data are expressed as mean ± SEM

 As shown in Figure 1, analysis of qRT-PCR results showed that the level of mRNA expression of NBC1 was increased in response to gastric distention (*p *< 0.01). NaHS significantly increased this level compared to D group (*p *< 0.01) whereas PAG treatment decreased NBC1 mRNA expression compared to the control level (*p *< 0.05).

**Fig. 2:**
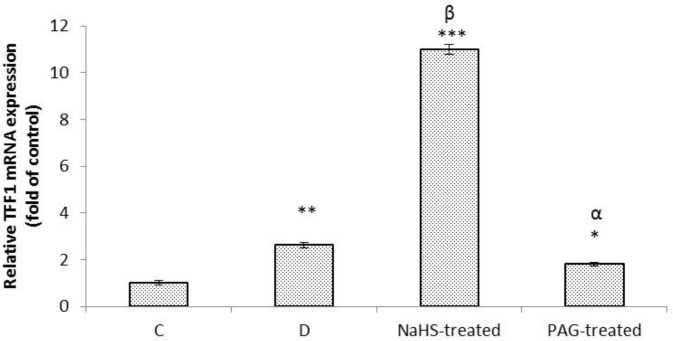
Effect of gastric distention alone and in combination with sodium hydrosulfate (NaHS) or propargilglycine (PAG) on mRNA expression of of TFF1 in gastric mucosa. NaHS-treated: Animals received H2S donor [NaHS], PAG-treated: Endogenously produduction of hydrogen sulfide was inhibited using selective CSE inhibitor (PAG). Analysis of qRT-PCR results showed that the level of mRNA expression of TFF1 in distention, NaHS-, and PAG-treated rats was higher than those in the control rats. *, ** and *** indicate signifcant difference compared to the control group (C) at *p *< 0.05, *p *< 0.01 and *p *< 0.001, respectively. ^α ^indicate significant decrease compared to distention group (D) at *p *< 0.05; ^β ^indicate significant increase compared to distention group at *p *< 0.001. Data are expressed as mean ± SEM

As shown in Figure 2, analysis of qRT-PCR results showed that the level of mRNA expression of TFF1 was increased in response to gastric distention (*p *< 0.01). NaHS significantly increased this level compared to distention group (*p *< 0.001). The PAG treatment significantly decreased the level of gene expression of TFF1 compared to distention (*p *< 0.05).

**Fig. 3:**
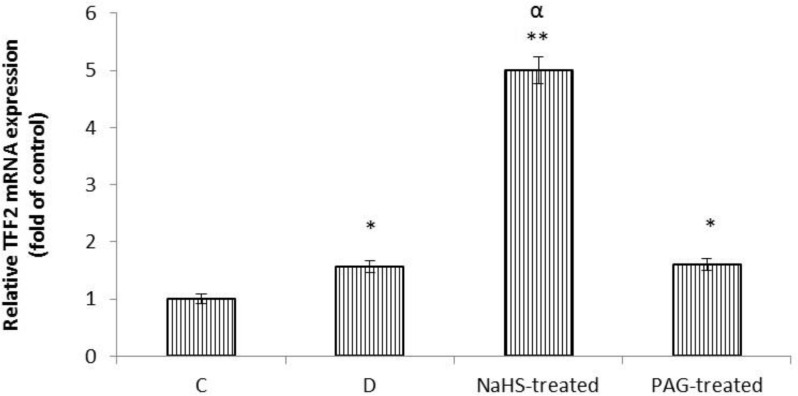
Effects of gastric distention alone and in combination with NaHS or PAG on mRNA expression of TFF2 in gastric mucosa. NaHS-treated: Animals received H2S donor (NaHS), PAG-treated: Endogenously produduction of hydrogen sulfide was inhibited using selective CSE inhibitor (Propargylglycine). Analysis of qRT-PCR results showed that the level of mRNA expression of TFF2 in distention, NaHS-, and PAG-treated rats was higher than those in the control rats. * and ** indicate signifcant difference compared to the control group (C) at *p *< 0.05, and *p *< 0.001, respectively. ^α^ indicates significant increase compared to distention group (D)  at *p *< 0.001. Data are expressed as mean ± SEM

As shown in Figure 3, analysis of qRT-PCR results showed that the level of mRNA expression of TFF2 was increased in response to gastric distention (*p *< 0.05). NaHS significantly increased the level of gene expression of TFF2 compared to distention group (*p *< 0.001). This level in PAG-treated rats was equal to distention group.

As indicated in Table 1, the total acid content of gastric contents in NaHS-treated rats was significantly lower than in D, and PAG-treated groups (*p *< 0.05 and *p *< 0.01, respectively).

**Table 1:** Effects of NaHS and propargylglycine (PAG) on gastric acid output in response to distention in rats. Data are shown as mean ± SEM

**Groups**	**Total acid content (µEqH** ^+^ ** per 90 min)**
**Control **	26.1 ± 1.8
**Distention group**	78.0 ± 7.2†
**NaHS-treated group**	53.5 ± 4.8*^a^
**PAG-treated group**	106.0 ± 6.0† b

* and † indicate significant differences compared to the control group at *p *< 0.01 and *p *< 0.001, respectively.

a and b indicate significant difference compared to distention group at *p *< 0.05 and *p *< 0.01, respectively.

## Discussion

 The findings of the present study showed that 1) The mucosal levels of mRNA expressions of NBC1, TFF1 and TFF2 were significantly increased in response to gastric distention and NaHS treatment; 2) Inhibiting the endo-genous production of hydrogen sulfide using selective inhibitor of CSE (PAG) decreased the expression levels of NBC1 and TFF1; and 3) The total acid content of gastric washout in NaHS-treated rats was lower than in Distention, and PAG-treated groups.

As shown in Figure1, the level of mRNA expression of NBC1 in distention group was significantly higher than in control rats. Little is known about the regulation of gastric bicarbonate secretion. Vagal stimulation and intraluminal acidity are the first and second powerful possible stimuli of gastric bicarbonate secretion.^16^ Moreover, an *in vitro* study showed that secretion of bicarbonate by fundic mucosa is dependent on luminal but not serosal Cl^-^ in frog.^9^^,^^17^ Physiologically, when the acid output increases, the luminal concentration of Cl^- ^also increases. To our best knowledge, there is no report on the effect of vagal stimulation and intraluminal acidity on gene expression of NBC1, however, the results of the present study showed that vagal activation and intraluminal acidity induced by gastric distention in addition to stimulating bicarbonate secretion (non-genomic effect) shown by previous report,^16^ upregulated the gene expression of NBC1.

Prostaglandin E_2_ (PGE_2_) has been shown to stimulate gastric bicarbonate release.^16^ NaHS has been shown to increase PGE_2_ and bicarbonate secretion in duodenum and gastric mucosa in rats.^5^^,^^18^ The results of present study also showed that NaHS was increased while propargylglycine decreased the level of mRNA expression of NBC1 compared to distention group.Therefore, these results together showed that exogenous and endogenous hydrogen sulfide in addition to stimulation of bicarbonate secretion as evidenced by previous report induced gene expression of NBC1 and implied that secretory effect of H_2_S on bicarbonate was partly mediated by upregulating the gene expression of NBC1.

According to Table 1, the total acid content of gastric washout in NaHS-treated rats was significantly lower than in distention and PAG-treated rats. This finding was in agreement with previous report.^6^ This increase might be due to 1) An increase in the level of gene expression of NBC1 in response to NaHS which it in turn led to increase in the level and activity of NBC proteins in gastric mucosa and 2) The anti-secretory effect of NaHS on distention-induced gastric secretion as reported by the previous study.^6^

In contrary to gastric distention, the upregulatory effect of H_2_S on mRNA expression of NBC1 was not dependent on the gastric acid because total acidity in distention and PAG-treated groups was higher than in NaHS-treated rats. Therefore, further studies need to be performed to describe the underlying mechanism of the upregulatory effect of H_2_S on NBC1.

The present results showed that the level of mRNA expressions of TFF1 and TFF2 were increased in response to gastric distention. G-cells in the pyloric glands of stomach primarily secrete a peptide hormone known as gastrin. Most important releasers of gastrin from antral G-cells are partly digested proteins, Ca^2+^, gastrin releasing peptide (GRP) and gastric distention.^19^ Gastrin has been shown to rapidly and potently induce mRNA expression of TFF1 and TFF2 in a AGS-G(R), and AGS-E gastric cancer cell lines, human Caucasian gastric adenocarcinoma, that expresses the CCK_B_ receptor.^20^^,^^21^ Also, protein expression of TFF2 has been reported to be increased in the gastric fundus in hypergastrinemic transgenic mice whereas decreased in gastrin-deficient mice.^21^ Therefore, these results suggested that an increase in gene expressions of TFF1 and TFF2 could be resulted from an increase in the plasma level of gastrin.

In this study, we for the first time showed that exogenous H_2_S upregulated mucosal mRNA expression of TFF1 and TFF2 in response to gastric distention. Peroxisome proliferator-activated receptor γ (PPARγ) has also been shown to protect the gastric mucosa through upregulation of mRNA expression of TFF2 in gastric epithelial cells.^22^ Therefore, this finding suggested that H_2_S in addition to inhibiting the gastric acid output and stimulating mucus and bicarbonate secretion as shown by the previous reports,^5^^-^^7^ act similar to PPARγ to protect the gastric mucosa against gastric acid and pepsin by inducing the gene expressions of TFF1 and TFF2 as demonstrated by the present results.

The another possible mechanism of upregulatory effect of hydrogen sulfide on mRNA expression of TFF1 and 2 could partly be mediated through its inhibitory effect on pro-inflammatory cytokines. Hydrogen sulfide has been shown to protect the gastric mucosa against ischemia-reperfusion induced-injury through decreasing the plasma, and mRNA levels of pro-inflammatory cytokines, IL-1β and TNF-α in rats.^23^ TNF-α and IL-1β have been demonstrated to reduce mRNA expressions of TFF1 and TFF2 in gastric epithelial cells.^24^^,^^25^ Therefore, these findings together implied that endogenous and exogenous H_2_S through inhibition of pro-inflammatory cytokines could induce gene expressions of TFF1 and TFF2.

In agreement with the present results, H_2_S has been shown to upregulate the gene expression of variety of mucosal protective proteins such as cyclooxygenases (1 and 2), calcitonine gene related peptide, and endothelial nitric oxide synthase (eNOS).^6^^,^^18^ It has been shown that H_2_S maintain the mucosal integrity of the rat stomach against ischemia-reperfusion injury by reducing the gene expressions, and plasma levels of pro-inflammatory cytokines [interleukin-1β and TNF-α] and at the same time by increasing the plasma levels of anti-inflammatory cytokines (TGF-β and IL-10).^23^ Moreover, another study showed that H_2_S through downregulating mRNA expressions of inflammatory factors, intercellular adhesion molecule (ICAM)-1, and lymphocyte function-associated antigen (LFA)-1, protect the gastric mucosa against NSAIDs-induced gastric ulcer in rats.^2^ Non-steroidal anti-inflammatory drugs (NSAIDs) have been demonstrated to induce the gastric lesions in rats through upregulation of the mRNA expression of inflammatory mediator, TNF-α.^2^ Upregulation of TNF-α in response to NSAIDs was inhibited by hydrogen sulfide pretreatment in gastric mucosa.^2^ Endogenous and exogenous hydrogen sulfide have been indicated to decrease acid output in response to gastric distention through reducing mRNA expression of α-subunit of gastric proton pump and at the same time inducing mRNA expression of cyclooxygenase-2.^6^ The above mentioned evidences show that the gastro-protective activity of hydrogen sulfide against physiologic [gastric acid]-, and non-physiologic irritants is in part mediated through upregulation of mRNA expressions of variety of mucosal protective proteins.

In conclusion, the findings of the present study showed that exogenous H_2_S upregulated the mucosal levels of mRNA expressions of NBC1, TFF1 and TFF2, and the mucosal levels of mRNA expressions of NBC1 and TFF1 were induced by endogenous hydrogen sulfide.
